# Polymorphic low-grade neuroepithelial tumor of the young and treatment of epilepsy: a case report

**DOI:** 10.3389/fonc.2026.1797276

**Published:** 2026-03-17

**Authors:** Guodong Li, Jibin Ren, Meimin Zheng, Xi Wu, Wei Li, Yongjun Wang, Nan Wu

**Affiliations:** 1 Department of Neurosurgery, Tianjin Children’s Hospital (Children’s Hospital of Tianjin University), Tianjin, China; 2 Tianjin Key Laboratory of Birth Defects for Prevention and Treatment, Tianjin Children’s Hospital (Children’s Hospital of Tianjin University), Tianjin, China; 3 Department of Radiology, Tianjin Children’s Hospital (Children’s Hospital of Tianjin University), Tianjin, China

**Keywords:** case report, ECoG, epilepsy surgery, MRI-PET, polymorphic low-grade neuroepithelial tumor of the young

## Abstract

**Background:**

Polymorphic low-grade neuroepithelial tumor of the young (PLNTY) is a rare central nervous system tumor. Clinical manifestations often start with seizures, and the lesions are often located in the superficial parts of the cerebral hemisphere, especially in the temporal lobe. Patients with PLNTY can be cured via surgical treatment, but whether the seizure can be controlled by simply removing the tumor through surgery still needs to be determined.

**Case description:**

The patient was a 6-year-old boy with clinical manifestations of recurrent epileptic seizures. Preoperative standardized antiepileptic drug treatment failed to control seizures. The patient’s electroencephalogram showed was right parietal and central regions is the main slow wave and spike slow wave emitting area, and Magnetic Resonance Imaging s(MRI) showed was structural abnormalities in the right parietal lobe cortex. After multidisciplinary preoperative evaluation at the epilepsy center of Tianjin Children’s Hospital, lesion enlargement resection was performed with the assistance of multimodal imaging and electrocorticography (ECoG) monitoring. There were no epileptic seizures during the 6-month follow-up after surgery.

**Conclusions:**

For this patient with PLNTY accompanied by epilepsy, surgical resection can be the first line of treatment. Meanwhile, a comprehensive multidisciplinary preoperative evaluation should be conducted. Additionally, appropriate enlargement and resection can effectively eliminate epileptic seizures.

## Background

Polymorphic low-grade neuroepithelial tumor of the young (PLNTY) is a rare central nervous system tumor that was first reported and named in 2016 (1). In 2021, it was added to the WHO classification of central nervous system tumors as a grade 1 pediatric low-grade glioma (2). PLNTY is more common in children and adolescents and clinically manifests as epileptic seizures. The lesions are usually located in the superficial part of the cerebral hemisphere and can occur in different lobes but are more commonly found in the temporal lobe. In patients with PLNTY, calcification and cystic changes are commonly observed on imaging. PLNTY is an indolent tumor that can be mostly cured through surgical resection (3). However, whether surgical resection alone can control epileptic seizures remains to be determined.

In accordance with the CARE guidelines, we report the case of a 6-year-old boy with a pleomorphic low-grade neuroepithelial tumor (PLNTY) and accompanying epilepsy, aiming to provide a comprehensive analysis of the diagnosis and treatment of PLNTY and associated epilepsy from the perspective of epilepsy surgery.

## Case presentation

The patient was a 6-year-old boy who had been seeking treatment at our epilepsy center for 4 years because of intermittent seizures. The symptom presentation consisted of episodic weakness, mainly affecting both legs, occurring 1–2 times a day and lasting from a few seconds to approximately 20 seconds per episode, with spontaneous resolution. After relief, mental and motor functions remained normal. The patient received three types of antiepileptic drugs orally before surgery (Okazepine 700mg/day, Levetiracetam 1g/day, Pirfenapyr 4mg/day), but the seizures showed no significant improvement. The patient’s mental state was good, and growth and development were normal. Auxiliary examinations included video electroencephalogram(VEEG) monitoring, MRI with an epilepsy sequence, and PET-CT.

**Figure 1:**
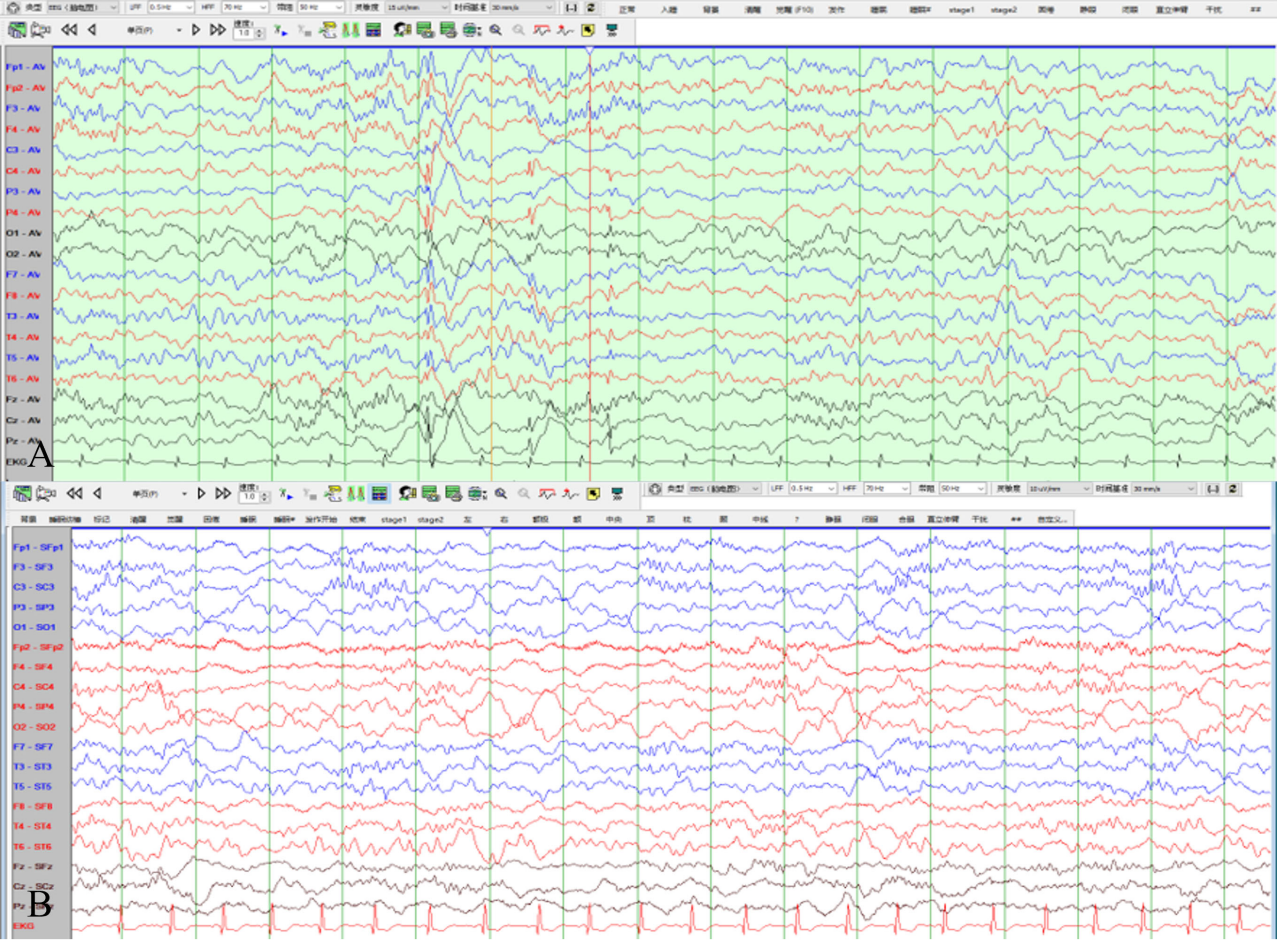
**(A)** During the interval between VEEG-recorded attacks before surgery, spike waves and slow spike waves are observed in the central, parietal, and midline regions of the right side, which may affect the left parietal and right temporal regions. **(B)** Postoperative VEEG shows diffuse wave activity around the original lesion area. (LFF:0.5Hz,HFF:70Hz,Voltage:15uV/mm, Sweep speed:30mm/s, the International 10–20 system was utilized.).

In VEEG, during the interval between VEEg-recorded attacks, spike waves and slow spike waves were observed in the central, parietal, and midline regions of the right side, which affected the left parietal and right temporal regions. Two seizure episodes were detected during the awake period, characterized by limb weakness and a backward tilt of the body when sitting. During the same period, EEG showed low-amplitude fast waves in the right parietal and central regions; low- to medium-amplitude sharp waves in the right frontal pole, frontal, and anterior middle temporal regions; and extensive medium- to high-amplitude diffuse waves and pointed diffuse waves mixed with spike waves in the bilateral central, parietal, and midline (Cz, Pz) regions. The amplitude gradually increased, and the frequency gradually slowed down, lasting for 19–20 seconds. (**Figure 1A, B**).

**Figure 2:**
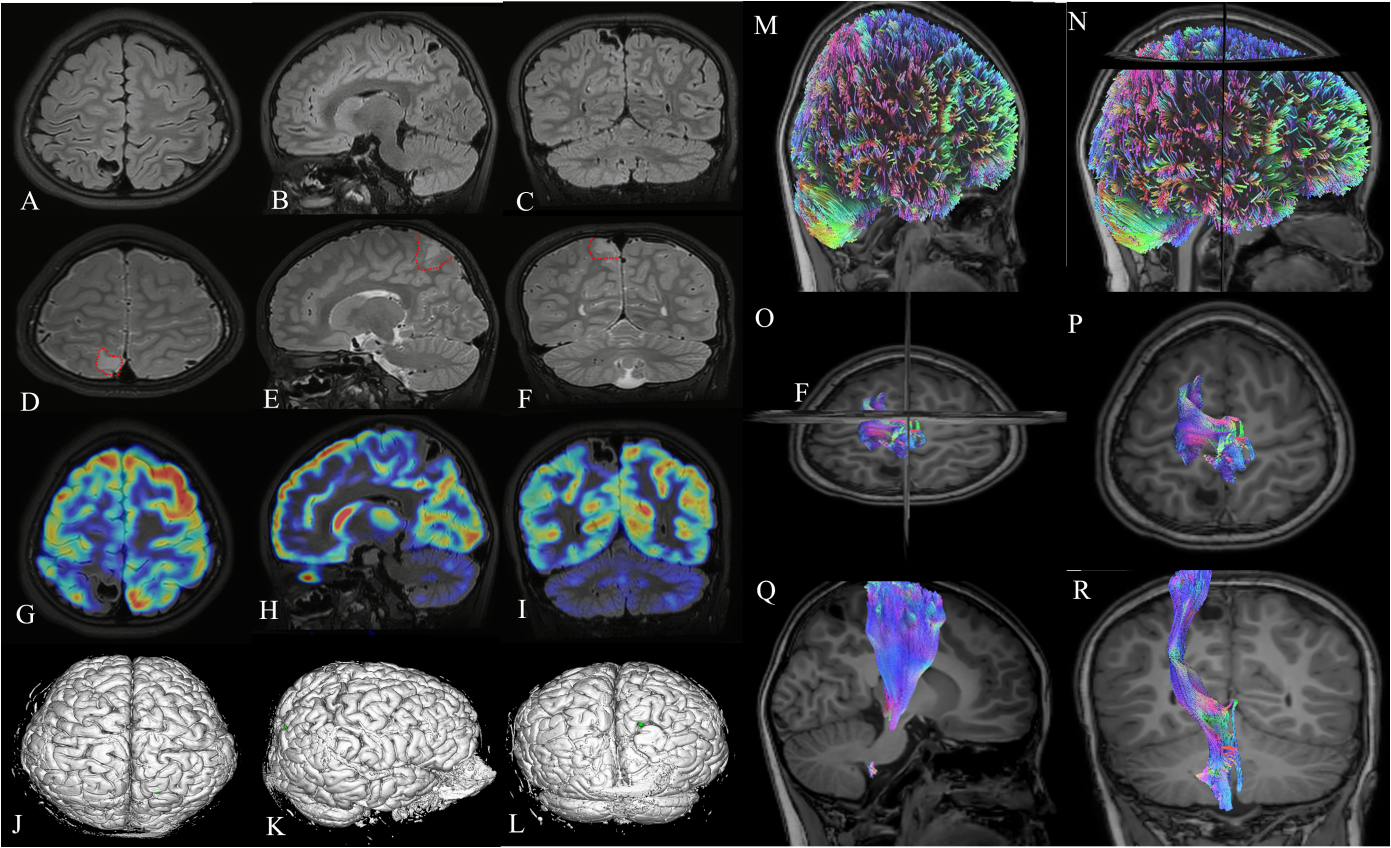
Imaging examinations of the patient: **(A–C)** MRI T2 FLAIR (axial, sagittal, and coronal); **(D–F)**, MRIT2 (axial, sagittal, and coronal), The salt and pepper sign around the tumor can be seen at the red dashed line; **(G–I)**, PET-MRI (axial, sagittal, and coronal); **(J–L)**, Stereoscopic imaging of brain tissue. Display of the parietal lobe lesion on DTI. **(M, N)** DTI before surgery. **(O–R)** There was no significant correlation between the corticospinal tract and the parietal lobe lesion area according to DTI. DTI, diffusion tensor imaging; MR, magnetic resonance.

MRI demonstrated patchy long T1 and long T2 signals(salt and pepper sign) in the right parietal lobe. T2-FLAIR and DIR images showed a slightly high signal surrounded by a low signal, with a size of approximately 15 mm × 15 mm × 12 mm. The lesion appeared to have expanded, and the adjacent cortex became thinner and locally discontinuous. During PET-CT, no FDG uptake was observed within the lesion, and there was a significant uneven decrease in FDG uptake in the adjacent cortex. (**Figures 2A-I**)

A multidisciplinary epilepsy center (neurology, neurosurgery, imaging, etc.) conducted a preoperative evaluation of the patient. Based on the patient’s clinical symptoms, VEEG monitoring, imaging findings, and the anatomical electrical clinical concept, the patient met the indications for epilepsy surgery and could undergo surgical treatment. We performed multimodal image fusion integrating MR-PET (**Figures 2G-I**), simulated 3D brain tissue (**Figures 2J-L**), and diffusion tensor imaging (DTI)(**Figures 2M-R**) before surgery to determine the extent of lesion resection.

**Figure 3:**
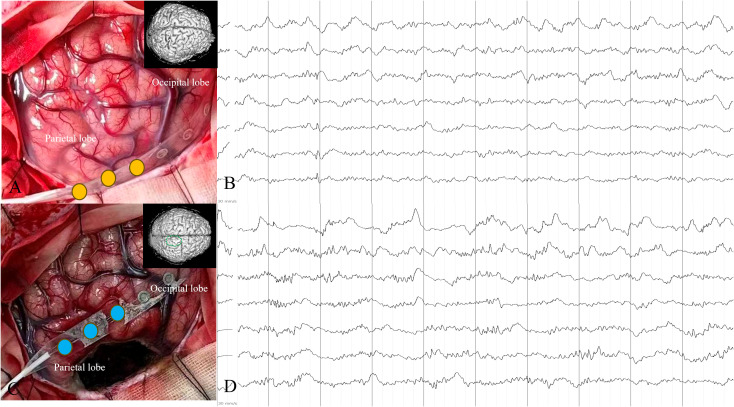
White matter electroencephalogram (ECoG) monitoring **(A, B)** shows scattered spike waves **(C, D)** in the right parietal lobe lesion area. Based on preoperative stereo-images of the brain tissue, surgical resection was planned. After surgical resection of the lesion, scattered diffuse waves were observed in the surrounding area.

After the induction of general anesthesia, a U-shaped craniotomy was performed on the right side. ECoG showed spike discharges in the epileptic lesion area, and the central area was protected. The parietal lesion was removed along the parietal cortex, and after resection, ECoG showed the disappearance of abnormal discharge (**Figure 3**). According to the preoperative plan, the tumor and surrounding area were removed, bleeding was stopped, the skull was closed, and surgery was completed. Postoperative treatment included anti-infection therapy, alleviation of cerebral edema, and prevention of bleeding.

Histopathology revealed tumor cell infiltration, growth, and polymorphic changes.

**Figure 4:**
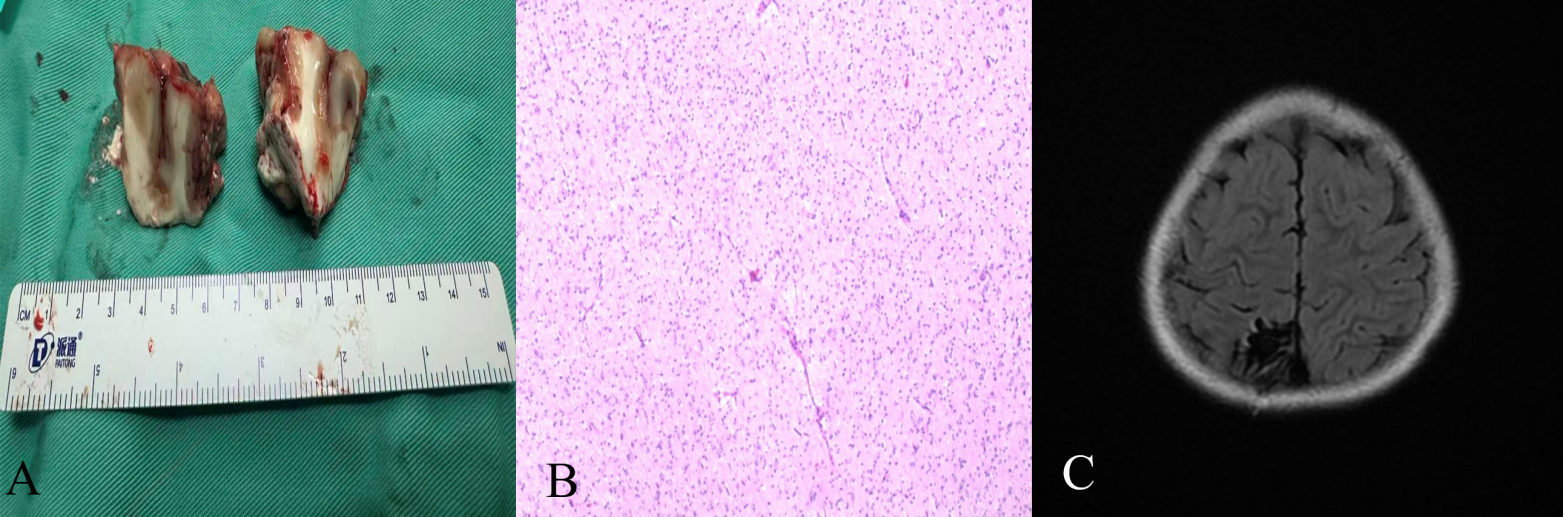
Pathological examination and postoperative imaging of the lesion. **(A)** Photo of surgical resection of the lesion, showing complete resection of the lesion. **(B)** Pathology (HE × 100). **(C)** Postoperative magnetic resonance imaging shows complete resection of the lesion.

Immunohistochemistry revealed the following: H3K27M (-), IDH1 R132H (-), NeuN (partially +), BRAF V-600E (-), EMA (±), P53 (focal ±), GFAP (+), P16 (partially +), NF (2F11) (interstitial +), Syn (partially +), INI-1 (+), Vimentin (partially +), Olig-2 (+), SOX10 (+), H3K27Me3 (+), S-100 (partially +), ATRX (+), CD34 (partially +), and a Ki-67 protein-positive expression index of 2%. The patient was diagnosed with PLNTY (WHO grade I/II). (**Figures 4A, B**)

On the second postoperative day, oral antiepileptic drugs were administered, and no seizures or complications were noted. Postoperative magnetic resonance (MR) imaging revealed complete resection of the lesion (**Figure 4C**). The patient was discharged on the 14th day after surgery.

The patient was seizure-free, had no functional impairments, and was followed up for 6 months after surgery.

## Discussion

PLNTY is a rare central nervous system tumor. Since Huse’s first report and naming, there have been only a few reports of PLNTY in the literature (1), the vast majority of which are individual case reports. When encountering central nervous system tumors that are difficult to diagnose clinically, the possibility of PLNTY should be considered.

Compared with other gliomas, the imaging features of PLNTY have the following characteristics: (1) The vast majority of PLNTY are located on the surface or superficial part of the cerebral hemisphere, mostly in the temporal lobe, with clear boundaries. Some have subcortical phenomena and a certain degree of invasiveness, and the tumor-occupying effect is not obvious; there is no peritumoral edema or only mild edema (4, 5). (2) According to the MRI signal manifestations of the lesion, T1WI may show low signal intensity, and T2WI may have high signal intensity, and in some cases, “salt and pepper signs” may be seen, often accompanied by calcification and cystic changes (6, 7). The “salt and pepper sign” on T2WI may be a characteristic feature of PLNTY, which may be attributed to granular calcification within the tumor. The mechanism underlying the formation of the “salt and pepper sign” may be related to calcification, the vascular void effect, and the distribution of different cellular components within the tumor. The calcified lesions within the tumor appear as low-signal punctate shadows on T2WI, while the vascular void effect is manifested as a tubular structure without signal, which contrasts with the high-signal tumor tissue and presents a “salt and pepper sign”. PLNTY is often accompanied by calcification and cystic changes. The occurrence of calcification may be related to metabolic abnormalities in tumor cells and changes in the local microenvironment. Cystic changes may occur due to ischemia, necrosis of tumor tissue, or the accumulation of fluid secreted by tumor cells. In enhanced scans, the degree of enhancement of PLNTY varies, and some tumors do not show significant enhancement. The MRI results of the patient are consistent with the imaging features of the vast majority of PLNTY cases mentioned above. On plain MR imaging, the lesion appeared slightly isointense on T1WI and T2WI sequences, without any occupying effect. There is no clear boundary between the lesion and normal brain tissue. Although the patient’s tumor diagnosis was clear, considering that their history of epilepsy was more than 4 years, the epilepsy center considered the symptomatology of focal seizures and the VEEG to indicate the presence of a pathogenic epilepsy network in the patient. Therefore, in preoperative planning, we also used PET-MRI image fusion technology to achieve the goal of treating the tumor while minimizing postoperative seizures.

PET-CT is generally used to evaluate the metabolic status of brain tumors and has significant advantages in terms of the degree of malignancy, scope, and reflection of tumor proliferation activity (8, 9). PET is also widely used for the preoperative evaluation of epilepsy surgery. The PET-CT results of this patient revealed no FDG uptake in the lesion, and FDG uptake in the adjacent cortex was significantly and unevenly reduced. This finding reflects not only the highly differentiated tumor characteristics of PLNTY but also the low PET-CT metabolism in patients with structural epilepsy lesions, which often reflects the potential epileptic characteristics of the surrounding area.

DTI is a commonly used imaging technique for preoperative surgical planning in neurosurgery (10, 11), and this technique was also applied in the preoperative planning of the present case. DTI plays an important role in displaying white matter fiber bundles and adjacent structures for surgical planning, especially in protecting related motor functions. It is widely used in clinical practice. In the present case, the lesion was located in the parietal lobe, close to the central cortex. Therefore, preoperative DTI–MRI fusion could guide surgical path planning and minimize surgical damage to the pyramidal tract. The patient’s postoperative function was good, and no obvious abnormalities were observed.

Histopathological examination is the gold standard for diagnosis, with immunohistochemical staining for CD34 (+), GFAP (+), and OLIG2 (+) being commonly observed. IDH1 R132H, EMA, NeuN, and neuroendocrine markers are often negative, and the MIB-1 labeling index is usually low (<1%–2%) (12). The abnormal expression of CD34 may be closely related to the proliferation, differentiation, and angiogenesis of tumor cells. The positive expression of CD34 helps to distinguish it from other low-grade neuroepithelial tumors, such as oligodendroglioma, which is usually CD34 negative, while the positive expression of CD34 in PLNTY has certain characteristics. Glial fibrillary acidic protein (GFAP) can also show varying degrees of positive expression in PLNTY. GFAP is a marker of astrocytes, and its positive expression suggests that tumor cells may have certain differentiation characteristics of astrocytes, but the expression intensity is usually lower than that of typical astrocytomas. The immunohistochemical results of the patient revealed GFAP (+), S-100 (+), and Syn (+), strongly suggesting that the tumor originated from astrocytes and multipotent neurons. The Ki-67 index of this patient was 2%, indicating a low proliferation index, which is consistent with the characteristics of an inert tumor. However, some studies have reported cases of difficult treatment control or disease recurrence, for which long-term follow-up and re examination are needed (13, 14). Some studies have shown that PLNTY has a BRAFV600E gene mutation, which can activate the mitogen-activated protein kinase (MAPK) pathway, leading to tumor cell proliferation (15, 16). Mutation of the BRAF gene may affect neural networks, leading to abnormal firing of various neurons at different locations, which may result in various epileptic seizures (14). The association between the BRAF V600E mutation and tumors deserves further attention in future mechanistic studies (17). In this case, unlike the concept of tumor neurosurgery, we gain the following insights from the perspective of epilepsy surgery:

First, inconsistencies may exist between tumor lesions and epileptic lesions.

The most common clinical manifestation of PLNTY is epilepsy, which is a type of long-term epilepsy-associated tumor (LEAT) (18, 19). With respect to patients with PLNTY and tumors with epilepsy, multiple studies have elucidated the lack of consistency between tumor lesions and epileptic lesions, but the pathological and physiological mechanisms involved remain unclear (20). Research has shown that incomplete tumor resection accompanied by cortical dysplasia is the main cause of postoperative epilepsy recurrence.

Epilepsy is a type of brain network disorder in which the connections between the brain network are primarily established via nerve fibers (21–23). The occurrence of epilepsy and the establishment of an epileptic network depend on the interaction between the tumor and surrounding tissues. From the perspectives of anatomy and physiology, the occurrence of epileptic seizures is based on the interrelationship of four “regions”, namely, the epileptic zone, seizure initiation zone, irritability zone, and clinical symptom generation zone. It is usually believed that tumor removal can achieve the goal of curing epilepsy (24). However, through the study of surgical procedures for patients with tumor-related epilepsy, it has been noted that removing only the tumor cannot effectively control epileptic seizures (25). Given that epilepsy is a network disease (26), the disappearance of epileptic seizures after tumor resection may be due to the surgical cutting off of the network connection between the tumor lesion and surrounding tissues (18, 27). However, why can epilepsy still recur after complete tumor resection? We believe this may be due to the presence of a previous epilepticy network, in which the epileptic zone is not limited to the tumor itself. Surrounding brain tissues can also serve as potential epileptic areas, a phenomenon particularly prominent in patients with a long history of epilepsy. Therefore, it is necessary to reasonably plan the resection range by combining multimodal imaging techniques in the treatment of tumor-related epilepsy.

Second, multidisciplinary diagnosis and treatment in epilepsy centers are crucial for the surgical evaluation of patients with tumor-related epilepsy.

In the surgical process, “total resection” of the lesion should not be limited to the tumor itself. Preoperative multimodal imaging is used to evaluate the epileptic area of the tumor. Studies have shown that the electrical conduction range in patients with epilepsy is usually greater than that of the original lesion area. MRI alone shows a relatively limited range of lesions, but compared with MRI, PET imaging results in a greater range of metabolic decline (28, 29). Therefore, integrating PET-MRI fusion with ECoG monitoring allows for extensive resection aimed at achieving complete resection of the tumor and associated epileptic focus. Therefore, relying solely on neurosurgery for preoperative evaluation is clearly not enough. Multidisciplinary collaboration models (including neurology and imaging departments) are crucial for improving the efficacy of surgical treatment (30). We adopted this preoperative evaluation mode for the present patient, and the surgery achieved expanded resection. The patient was followed up for 6 months after surgery, and he experienced no seizures.

## Conclusion

In summary, relying solely on structural imaging, such as MRI, for the preoperative planning of patients with tumor-related epilepsy may allow complete tumor resection. However, the treatment of tumor-related epilepsy still requires the combination of multimodal imaging to plan the surgical resection range. Moreover, a comprehensive multidisciplinary evaluation should be conducted before surgery rather than relying solely on neurosurgical assessment to determine surgical treatment. Multimodal imaging and intraoperative ECoG are crucial for successful surgery. Intraoperative total tumor resection and appropriate enlargement resection can effectively control epileptic seizures.

## Data Availability

The raw data supporting the conclusions of this article will be made available by the authors, without undue reservation.

## Glossary

PLNTY: Polymorphic low-grade neuroepithelial tumor of the young
MRI: Magnetic Resonance Imaging
PET-MRI: Positron Emission Computed Tomography- Magnetic Resonance Imaging
EEG: electroencephalogram
Ecog: electrocorticography
